# Supplementary material for: HIVprotI: an integrated web based platform for prediction and design of HIV proteins inhibitors
Source: J Cheminform. 2018 Mar 9;10:12. doi: 10.1186/s13321-018-0266-y (PMC5845081; doi:10.1186/s13321-018-0266-y)
Supplement: Supplementary file 2 — Additional file 2. Source code of HIVProtI web server. [file 13321_2018_266_MOESM2_ESM.zip › HIVprotI_Source-code/hivprotI/jsme/test_depict_many_smiles_in_table.html]

SMILES performance test


# JSME in depict mode initialized with a SMILES- Performance test using 100 PubChem smiles.

Click the button to create a TABLE with 100 SMILEs depictions.

Try it
